# Phenotypic Impact of Rare Potentially Damaging Copy Number Variation in Obsessive-Compulsive Disorder and Chronic Tic Disorders

**DOI:** 10.3390/genes13101796

**Published:** 2022-10-05

**Authors:** Behrang Mahjani, Rebecca Birnbaum, Ariela Buxbaum Grice, Carolina Cappi, Seulgi Jung, Marina Natividad Avila, Abraham Reichenberg, Sven Sandin, Christina M. Hultman, Joseph D. Buxbaum, Dorothy E. Grice

**Affiliations:** 1 Seaver Autism Center for Research and Treatment, Icahn School of Medicine at Mount Sinai, New York, NY 10029, USA; 2 Division of Tics, OCD and Related Disorders, Icahn School of Medicine at Mount Sinai, New York, NY 10029, USA; 3 Department of Psychiatry, Icahn School of Medicine at Mount Sinai, New York, NY 10029, USA; 4 Department of Medical Epidemiology and Biostatistics, Karolinska Institutet, 171 77 Stockholm, Sweden; 5 Department of Genetics and Genomic Sciences, Icahn School of Medicine at Mount Sinai, New York, NY 10029, USA; 6 The Mindich Child Health and Development Institute, Icahn School of Medicine at Mount Sinai, New York, NY 10029, USA; 7 Friedman Brain Institute, Icahn School of Medicine at Mount Sinai, New York, NY 10029, USA; 8 Department of Neuroscience, Icahn School of Medicine at Mount Sinai, New York, NY 10029, USA

**Keywords:** obsessive-compulsive disorder, potentially damaging variation, copy number variation, chronic tic disorders, Tourette syndrome

## Abstract

Background: Recent studies report an important—and previously underestimated—role of rare variation in risk of obsessive-compulsive disorder (OCD) and chronic tic disorders (CTD). Using data from a large epidemiological study, we evaluate the distribution of potentially damaging copy number variation (pdCNV) in OCD and CTD, examining associations between pdCNV and the phenotypes of probands, including a consideration of early- vs. late-diagnoses. Method: The Obsessive-Compulsive Inventory-Revised (OCI-R) questionnaire was used to ascertain psychometric profiles of OCD probands. CNV were identified genome-wide using chromosomal microarray data. Results: For 993 OCD cases, 86 (9%) were identified as pdCNV carriers. The most frequent pdCNV found was at the 16p13.11 region. There was no significant association between pdCNV and the OCI-R total score. However, pdCNV was associated with Obsessing and Checking subscores. There was no significant difference in pdCNV frequency between early- vs. late-diagnosed OCD probands. Of the 217 CTD cases, 18 (8%) were identified as pdCNV carriers. CTD probands with pdCNV were significantly more likely to have co-occurring autism spectrum disorder (ASD). Conclusions: pdCNV represents part of the risk architecture for OCD and CTD. If replicated, our findings suggest pdCNV impact some OCD symptoms. Genes within the 16p13.11 region are potential OCD risk genes.

## 1. Introduction

Obsessive-compulsive disorder (OCD) is characterized by intrusive and unwanted thoughts, images, or urges (obsessions), as well as repetitive behaviors or mental rituals (compulsions) that typically function to reduce the distress associated with obsessions. OCD can cause significant and severe impairment; a better understanding of its etiological factors may inform the development of more effective treatment interventions.

Heritability for OCD is estimated at around 30–60% [1], indicating a significant genetic component comprises OCD risk. Although the literature is mixed [2,3,4,5], some reports have established a bimodal distribution for the age of OCD onset, with childhood- or early-onset (peak one) occurring during pediatric years and adult- or late-onset (peak two) occurring during young adulthood [6]. About 25% of OCD cases are symptomatic by early teenage years, and the mean age of onset for OCD is 19.5 years [7].

Research suggests that patients with childhood-onset OCD have different clinical and biological profiles than those with adult-onset OCD, and that childhood-onset OCD is associated with less favorable clinical outcomes [8]. There is also evidence of a further increased familial risk associated with childhood-onset OCD; analysis from childhood-onset samples estimates heritability of OCD to be around 45–61% for obsessive-compulsive symptoms [9], compared to heritability estimates of around 30–40% for adults [9,10,11].

Given the high heritability of OCD and the possibility that there is a genetically-defined differential profile specific to onset age, gene discovery may elucidate the etiology of OCD and provide a biological context for the development of novel treatments. To date, most genetic studies of OCD have primarily focused on the impact of common heritable variation on risk. However, there is increasing evidence implicating the role of rare variants in risk for OCD [12,13].

Importantly, OCD often presents alongside other co-occurring disorders, which tend to be related to OCD—in terms of both phenotypic presentation and underlying physiology. Around 30% of individuals with OCD also have history of a co-occurring diagnosis of Tourette syndrome, a chronic tic disorder [1,14,15,16,17,18,19,20], partly due to shared genetic risk [21,22]. Tourette syndrome is perhaps the most well-known diagnosis within the class of chronic tic disorders—a group of childhood-onset neurodevelopmental disorders that are defined by motor and/or vocal (phonic) tics persisting for at least one year. The diagnosis of different tic disorder subtypes is determined by the type of tic(s) present (motor, vocal, or a combination of the two) and the duration of symptoms (less than 12 months or greater than 12 months, i.e., persistent/chronic). Diagnostic criteria for Tourette syndrome, for instance, is defined by the presence of at least two motor tics and at least one phonic tic that onset before age 18 and have been present at least over the span of one year. In cases when only motor tics, or only vocal tics, are present, the diagnosis of persistent (chronic) motor tic disorder, or vocal tic disorder, is applied. For this study, we define Tourette syndrome and related chronic tic disorders jointly as chronic tic disorders (CTD). CTD, by this definition, has an estimated heritability varying from 21% to 77% depending on ascertainment, diagnostic instruments, and study design [23]. 

Copy number variation (CNV) describes a common form of structural variation and source of genetic diversity, in which a conserved genomic sequence presents in different “counts” (copy number) between individuals, frequently resulting in altered gene dosage. Gene dosage imbalances caused by rare structural variations (e.g., CNV) have been identified as a risk factor for several neurodevelopmental disorders (e.g., autism spectrum disorder, intellectual disability, and schizophrenia) [24,25], with many CNV having pleiotropic effects across disorders [26,27]. Several studies have determined that both de novo and inherited rare CNV are associated with the risk of developing OCD [25,28,29,30,31,32,33,34,35] and CTD [31,36,37,38,39,40].

Despite the existence of some reports, the population prevalence and characterization of CNV are understudied for OCD and CTD. Previous CNV analyses for OCD and CTD have often relied on *convenience samples*–a type of nonprobability sample in which study participants are included in the sample seeking care in a specialty clinic. A convenience sample is unlikely to be representative of the overall affected population, and conclusions drawn from convenience samples may not be directly applicable to a broader, non-selected population with the disorder. In addition, the population frequency of potentially damaging CNV (pdCNV), associated with early- or late-onset OCD cases within the same sample population, has not yet been reported. It is possible that a higher rate of pdCNV in early-onset OCD patients, compared to late-onset, could partially explain the less favorable outcomes associated with early-onset OCD. 

We previously developed and curated a large population-based cohort, called EGOS (Epidemiology and Genetics of Obsessive-Compulsive Disorder and Chronic Tic Disorders in Sweden) [41], to enhance etiological discovery in OCD and CTD. Here, we describe the analysis of pdCNV in OCD and CTD using data from the EGOS study. By incorporating robust and relatively unbiased phenotype data obtained from the Swedish national registers, we examine and test for possible associations between phenotypes of individuals diagnosed with OCD or CTD with, or without, pdCNV. Identification of pdCNV associated with OCD and CTD will yield a more detailed understanding of the pathobiology of these complex conditions while also highlighting potentially common sources of risk for OCD, CTD, and other neurodevelopmental disorders. 

## 2. Materials and Methods

### 2.1. Study Population

In this study, we used data from study participants in EGOS, a large ongoing population-based cohort study in Sweden [41]. Ethical approval was obtained from the Institutional Review Board at the Icahn School of Medicine at Mount Sinai, in New York, NY, USA, and the Regional Ethical Review Board in Stockholm, Sweden. 

The EGOS study cohort is composed of patients with a diagnosis of either OCD or CTD from the Swedish National Patient Register. All individuals living in Sweden and who were at least 16 years old in 1997 with a clinical diagnosis of OCD or CTD in the Swedish National Patient Register were eligible for inclusion in the source population. Within this source population, individuals who had at least two clinical diagnoses of OCD or two clinical diagnoses of CTD (diagnoses are entered into the register each time an individual attends a mental health care visit) were selected to participate in the molecular study. The Swedish translation of Obsessive-Compulsive Inventory-Revised (OCI-R) was provided as a web questionnaire to all participants. For more details about the EGOS cohort, see Mahjani et al., 2020 [41].

Information about sex, age at the time of diagnosis, dates of admissions and discharges, and psychiatric diagnostic codes [using the International Classification of Diseases (ICD), 10th revision (ICD-10)], were extracted from the Swedish National Patient Register. The psychiatric diagnoses were determined by a psychiatrist in a specialty care setting; then, diagnoses were registered using ICD codes. We used the date of the first psychiatric visit that led to the diagnosis of OCD or CTD as the age of diagnosis.

In this study, our analytic cohort consisted of data from 1249 affected individuals within EGOS: 1108 with OCD, 241 with CTD, and 100 diagnosed with both OCD and CTD. Around 88% of individuals were of European ancestry. 

### 2.2. CNV Identification

CNV calls were generated from 1249 samples genotyped on the Illumina Infinium Global Screening Array (GSA) by CNVision [42] using hg19 genomic coordinates. Sample-based quality control was based on the default setting of CNVision (genotype call rate > 95%, B Allele Frequency drift ≤ 0.01, |waviness factor| ≤ 0.05, log R ratio SD ≤ 0.28) [42]. Adjacent CNV calls were merged if the gap between them was ≤20% of the total length.

We focused on CNV found by both QuantiSNP and PennCNV algorithms. Then, we removed CNV that (1) failed quality control; (2) were categorized as deletion and duplication at the same time by two algorithms; (3) were non-genic; or (4) were in pericentromeric regions. 

We defined a CNV as rare if it had: (1) less than 50% reciprocal overlap by CNV with population frequency ≥1% in the Database of Genomic Variants (version 10), and size larger than 50 Kb; or (2) pCNV ≤ 1 × 10^−9^. CNVision’s pCNV parameter estimates the probability of a true CNV, based on per-SNP variability of Log R Ratio and the number of SNPs consistent with a CNV based on B Allele Frequency [42]. pCNV ≤ 1 × 10^−9^ indicates more than 95% confidence for de novo prediction. 

We called a rare CNV *potentially damaging* if it satisfied one or more of the following conditions: (1) if the CNV occurred within a locus associated with known genomic disorders curated by ClinGen and/or DECIPHER (as noted below); or (2) if the CNV was larger than 500 Kb and included one or more coding exons, similar to previous studies of OCD [31,34]. 

The list of known genomic disorders was derived from ClinGen [43] and DECIPHER [44]. We merged the ClinGen list with the list from ClinGen Dosage Sensitivity Curation Page [45] and chose the regions with sufficient evidence of haploinsufficiency and/or triplosensitivity (scores of three; sufficient evidence supporting dosage sensitivity). For DECIPHER, we excluded those with GRADE III (GRADE I: pathogenic anomaly, GRADE II: likely pathogenic anomaly, GRADE III susceptibility locus). For regions with discrepant classifications between the two databases, we used the ClinGen classification. The final list included 95 regions (Table S1) [46].

All pdCNV was manually curated by visual inspection using Illumina genomestudio v2.0 software (and confirmed with the cnvPartition CNV Analysis Plugin v3.2.1.

### 2.3. Severity of OCD Symptoms

OCI-R is a self-report questionnaire designed to assess the severity and type of symptoms of OCD [47]. The OCI-R measures six dimensions/subscores of OCD symptoms labelled as: Ordering, Obsessing, Checking, Washing/Contamination, Hoarding, and Neutralizing, using a 5-point scale from not at all (0 points) to extreme (4 points). It also has a total score, which is the sum of the subscores of all items. We used the OCI-R total score at the time of enrollment to measure the severity of OCD in the OCD probands. Given that our source population was sampled from those in specialized psychiatric care, we anticipate that most individuals received some treatment between their oldest diagnosis date and the date they completed the OCI-R questionnaire (we refer to this as the “time difference”). We have previously analyzed the OCI-R data from the EGOS cohort and shown that it had adequate psychometric properties [14]. In addition, we have shown that the time difference could explain the smaller OCI-R scores in our data compared to other studies [14].

### 2.4. Psychiatric Co-Occurring Conditions

The Swedish National Patient Register includes the ICD code for the primary diagnosis and up to thirty ICD codes for non-primary diagnosis, for each healthcare visit. To increase diagnostic specificity, we consider an individual to have a co-occurring condition if the condition is documented as the primary diagnosis, or at least twice as a non-primary diagnosis at two different time points.

We extracted information for the following variables using ICD-10 codes: attention-deficit/hyperactivity disorder (ADHD), anxiety disorders (phobic anxiety disorders, F40; and other anxiety disorders, F41), autism spectrum disorder (ASD), bipolar disorder, borderline personality disorder, eating disorders (F50), major depression, and schizophrenia.

### 2.5. Statistical Analysis

To identify co-occurring psychiatric conditions associated with OCD probands who carried pdCNV, we used a logit model in which the carrier status of the pdCNV was the dependent variable (carrier or not a carrier), and the covariates were sex and co-occurring psychiatric conditions (see Section 2.3 for the list of co-occurring psychiatric conditions). We reported the resulting odds ratio (OR), P-values, and 95% confidence intervals (CI) for the OR after adjusting for the sex variable. 

To determine the association between OCI-R scores and pdCNV status, we used linear regression. We adjusted for sex, time difference, and interaction between sex and time difference. We reported the resulting points estimate, 95% CI, and marginal means for pdCNV carriers and non-carriers.

## 3. Results

### 3.1. Demographic Data

Samples for 993 OCD and 217 CTD probands passed quality control (Table 1). Ninety-one individuals were diagnosed with OCD and CTD. Overall, 63% of the OCD probands were female, and females had a higher age of diagnosis (Table 1). The average age of OCD diagnosis within this group was 21.9 (SD = 7), and 31% of participants were diagnosed before age 18. Of the CTD probands, 36% were female (Table 1).

### 3.2. Genetic Findings

In total, we found 2,856 rare CNV among OCD probands, 604 rare CNV among CTD probands, and 238 rare CNV among OCD probands with CTD, representing a mean of 2.81 (SD = 2.15) rare CNV events per individual for OCD, 2.76 (SD = 1.54) for CTD, and 2.62 (SD = 1.60) for OCD with CTD. For the OCD probands, we found 89 pdCNV in 86 individuals (9%; Table 2 and Table S2). Nine OCD probands had pdCNV (1%) corresponding to known genomic disorders (Table 3 and Table S1), including 1q21.1 deletion (*n* = 1), 5q35 duplication (*n* = 1), 15q25.2 deletion (*n* = 1), 16p11.2 deletion (*n* = 1), 16p13.3 deletion (*n* = 1), 22q11.2 deletion (*n* = 2), and Xq28 duplication (*n* = 2).

Recurrent CNVs are the same size, have the same breakpoints, and are connected to certain locus control regions. In our study, two individuals had 16p13.11 duplication (BP2-BP3; includes MYH11), making this the most frequent recurrent pdCNV observed. However, our a priori criteria required consensus between Decipher and ClinGen, so the 16p13.11 duplication was not included in the primary downstream analyses (Decipher included 16p13.11 duplication in the CNV syndromes lists, while ClinGen had a triplosensitivity score of two for this region, suggesting some evidence for dosage pathogenicity; we, however, required a score of three). One individual with both OCD and CTD had a recurrent 15q25.2 deletion.

For CTD, we found 19 pdCNV events in 18 individuals (8%; Table 2). Three CTD probands (1%) had pdCNV that occurred within a locus associated with known genomic disorders. One individual with both OCD and CTD had a recurrent 15q25.2 deletion. For OCD with CTD, we found nine pdCNV events in eight individuals (9%). 

Numbers of pdCNV were not significantly higher in probands with an earlier age of OCD diagnosis (<18 years of age) compared to those with later age of OCD diagnosis (≥18 years). Similarly, there was no significant difference in pdCNV found in CTD probands as a function of late vs. early diagnosis. 

### 3.3. Co-Occurring Psychiatric Conditions in the Probands

Among OCD probands, anxiety disorders (28%), major depression (20%), and ADHD (7%) were the most common co-occurring psychiatric conditions (Table 4). Among CTD probands, ADHD (30%), anxiety disorders (19%), and ASD (14%) were the most common psychiatric co-occurring conditions. 

Among co-occurring psychiatric conditions with OCD, OCD with ASD (15%) and OCD with bipolar disorder (13%) had the highest rate of pdCNV (Table 4). Among co-occurring psychiatric conditions with CTD, CTD with ASD (21%) and CTD with major depression (8%) had the highest rate of pdCNV (Table 4). Among co-occurring psychiatric conditions with OCD and CTD, ASD (21%) had the highest rate of pdCNV (Table 4).

We examined the rate of pdCNV among probands with at least one psychiatric co-occurring condition compared with those without, to see whether there was statistically significant elevation for those with co-occurring psychiatric conditions (Table 5), but found no meaningful difference between the two groups.

However, there was a significantly higher rate of co-occurring ASD in CTD probands who were pdCNV carriers (Table 6). This difference was not seen with respect to the other psychiatric conditions (ADHD, anxiety disorders, bipolar disorder, borderline personality disorder, eating disorders, major depression, and schizophrenia) in either OCD or CTD probands carrying pdCNV (Table 6).

### 3.4. Severity of OCD among Carriers of pdCNV

OCI-R data were available for 580 (58%) individuals with OCD. Overall, 51 (9%) of 580 were carriers of pdCNV. The rate of missing values for the OCI-R variable was not significantly higher among carriers of pdCNV compared to non-carriers (41% vs. 42%). The average time difference in this group (the difference between an individual’s oldest diagnosis date and the date the individual completed the OCI-R questionnaire, measured in years) was 7 years, with a range from 3 to 17 years. Therefore, we removed all individuals with a time difference larger than 13 years (16 non-carriers of pdCNV and two carriers of pdCNV) to decrease the estimation bias.

**Figure 1:**
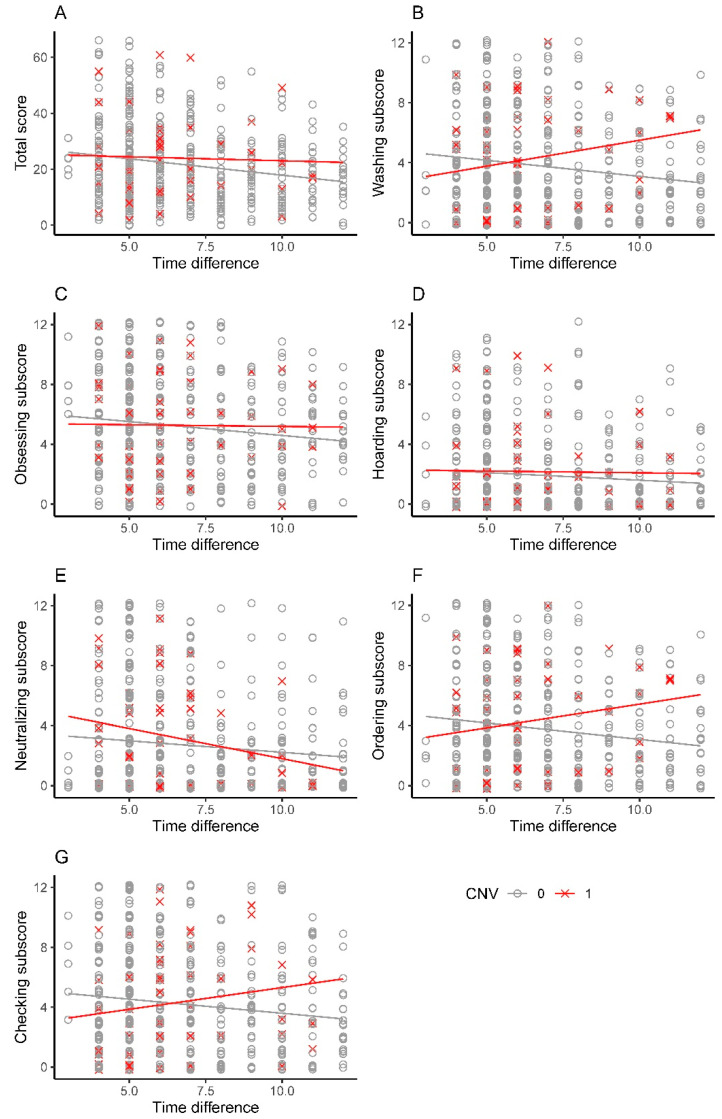
OCI-R score over the difference between an individual’s oldest diagnosis date in the register and the date the individual completed the OCI-R questionnaire, measured in years (time difference). (**A**) OCI-R total score, (**B**) OCI-R Washing subscore, (**C**) OCI-R Obsessing subscore, (**D**) OCI-R Hoarding subscore, (**E**) OCI-R Neutralizing subscore, (**F**) OCI-R Ordering subscore, (**G**) OCI-R Checking subscore. To visualize the overlapping points, we added a small random noise (jitter) to the plots.

We observed that the Obsessing, Hoarding, and Neutralizing subscores decreased over time for both carrier and non-carriers of pdCNV (Figure 1). However, while the Washing, Ordering, and Checking subscores decreased over time for non-carriers of pdCNV, they increased for carriers of pdCNV. The OCI-R total score was not significantly associated with pdCNV status after adjusting for sex, time difference, and interaction between time difference and pdCNV status (Table 7). However, the OCI-R subscores for the Ordering and Checking dimensions were significantly associated with pdCNV status (Table 7).

## 4. Discussion

We analyzed data from a large population-based epidemiological study in Sweden to evaluate the population characteristics of potentially damaging copy number variation in OCD and CTD. We identified potentially damaging variation in 9% of the OCD probands and 8% of the CTD probands, lower than other neurodevelopmental such as ASD [46]. The rate of pdCNV occurring within loci associated with known genomic disorders in our analysis was ~1%, somewhat lower than that reported in prior studies (1.5–3%) [31,33]. The 1q21.1 deletion, 16p13.3 deletion, and 16p13.11 deletion and duplication, observed in the EGOS cohort, were previously reported in individuals with OCD and CTD (Table 3) [25,31,33,48]. 

In our study of the EGOS cohort, the most frequent CNV was at the 16p13.11 region; we identified two duplications and one deletion that were observed in both OCD and CTD. The 16p13.11 deletion is a well-known genomic variant associated with multiple neurodevelopmental disorders such as anxiety disorders, ASD, epilepsy, and learning difficulties [25,49]. 

Importantly, multiple studies have reported the 16p13.11 duplication in OCD and CTD probands [25,31,33]. However, ClinGen listed 16p13.11 duplication with a triplosensitivity score of two due to non-specific clinical presentation associated with this region and the conflicting evidence of enrichment within the clinical population. The 16p13.11 CNV was the most significant finding from a prior study of OCD and CTD, in which four deletions and two duplications were observed in OCD (n = 1613) and one deletion was observed in Tourette syndrome (n = 1086) [31]. The same CNV was also identified in one individual with pediatric OCD by Gazzellone et al. (n = 307) [33] and also in one individual by Zarrei et al. (n = 222) [25]. Further studies are warranted to investigate dosage pathogenicity of 16p13.11 duplication and investigate its clinical features. One of the OCD patients with 16p13.11 in the EGOS cohort had co-occurring major depression and bulimia nervosa. 

There are more than 30 brain-expressed genes within the 16p13.11 locus. In this region, NDE1 and miR-484 are the two of the genes that are considered major contributors to the risk of neurodevelopmental disorders [50,51,52]. NDE1 (Nuclear Distribution Element 1) is highly expressed in developing brain and is associated with cortical malformations [53]. The central nervous system function of miR-484 is less well-studied; however, in a mouse model recapitulating 16p13.11 duplications (which has a hyperactivity phenotype), it has been shown that miR-484 promotes neurogenesis by inhibiting protocadherin-19 [53]. Future studies examining monosomy and trisomy of NDE1 and miR-484 in cell and animal models may provide insights into the pathobiological processes that contribute to OCD risk.

More broadlu, in 16p13.11 recurrent microdeletion/microduplication region (neurocognitive disorder susceptibility locus), Table S1, we observed eight brain-expressed protein-coding genes, including C16orf45, KIAA0430 NDE1, MYH11, FOPNL, ABCC1, ABCC6, and NOMO3. C16orf45, KIAA0430, and NDE1 had the highest expression level in different brain tissues. C16orf45 had the highest expression in the frontal cortex (median = 155), and KIAA0430 and NDE1 had the highest expression in the cerebellar hemisphere (median = 57 and median = 28, respectively).

Individuals with OCD can be at increased risk of cardiovascular diseases [54,55], which may be further influenced by CNV status. In a population-based, sibling-controlled cohort study in Sweden, individuals with OCD had a moderately increased risk of any cardiovascular disease with adjusted hazard ratios of 1.25 (95% CI, 1.22–1.29) [54]. Interestingly, a significant risk of cardiovascular disease has been reported in individuals with 16p13.11 duplication [56].

We identified a 15q25.2 deletion in one individual in the EGOS cohort, who was diagnosed with both OCD and CTD at the age of 14. 15q25.2 deletion has been previously reported in a patient with throat clearing/vocal tics, OCD, ADHD, and anxiety [57]. 15q25.2 is also commonly reported among individuals with ASD [58]. In fact, the individual in our cohort with 15q25.2 deletion was also diagnosed with ASD, supporting a pleiotropic role for this CNV. 

In the EGOS cohort, 3% of the OCD probands had a co-occurring bipolar disorder. 13% of OCD probands with co-occurring bipolar disorder were carriers of pdCNV, including one individual with 16p13.3 deletion. Bipolar disorder is a common co-occurring condition with OCD, reported in approximately 3% to 20% of patients with OCD [14,59,60]. In our recent study of OCI-R scores in the EGOS cohort, the total OCI-R score for individuals with OCD and bipolar disorder was significantly higher than individuals with OCD without any co-occurring psychiatric condition (*p*-value < 0.01) [14].

In the EGOS cohort, we did not observe a significantly higher overall OCD severity (measured by OCI-R total score) in the carriers of pdCNV compared to non-carriers, which could be due to the large standard deviation of the OCI-R scores, or variable expressivity/incomplete penetrance of the pdCNV. However, the subscores for Ordering and Checking were significantly associated with pdCNV status (Table 7). Interestingly, in our recent study of EGOS data, we observed a significantly higher score of Obsessing in individuals with OCD and at least one additional co-occurring psychiatric condition compared to individuals without any (*p*-value < 0.01) [14]. 

We observed that Ordering and Checking subscores decreased over time for non-carriers of pdCNV, likely due to a treatment effect, but increased for carriers of pdCNV (Figure 1). These results suggest that ordering and checking OCD symptoms, measured using the OCI-R, were more likely resistant to treatment among pdCNV carriers. However, other factors could also affect the OCI-R subscores over time; for example, linear trajectories could be impacted by small numbers of individuals at certain time points. Longitudinal studies are required to investigate these results more in-depth. This finding, if replicated, could accelerate research into biomarkers and novel treatments for OCD subtypes. In future studies, it will be important to investigate to what extent the joint effect of rare genetic variation, inherited and de novo, and common variation affects the severity of OCD symptoms. 

Age of symptom or disorder onset was not available for this study; only the age of OCD or CTD diagnosis was accessible. Early-diagnosed individuals had an early onset of OCD or CTD; however, late-diagnosed individuals could have had an early onset of OCD or CTD but were diagnosed later in their lives. Interestingly, the rate of pdCNV was not significantly different between early- and late-diagnosed OCD probands, suggesting that pdCNV, even if they may contribute to OCD phenotype, are not strongly related to onset. 

The present study has several strengths and some limitations. (1) The EGOS cohort is a population-based sample that reduces the bias in the estimators of population quantities of interest. In particular, we could determine the frequency of pdCNV in this population-based sample. (2) All individuals had at least two clinical diagnoses of OCD by a psychiatrist, which minimizes the likelihood of misdiagnosis. (3) While the Swedish National Patient Register is considered a robust and reliable source for research, utilizing ICD diagnostic criteria, the register lacks information about the individuals who do not seek clinical services at all or are treated solely at primary care practice. Hence, our sample may have over-represented more severe cases. (4) We did not have de novo information to aid in the classification of pdCNV. Inheritance of a genetic variation is used as an important determinant of pathogenicity and some variations, if inherited, might not be damaging. 

## 5. Conclusions

We observed that around 1 in 12 OCD and CTD probands in our study were carriers of potentially damaging CNV. CNV 16p13.11 is emerging as a recurrent finding in OCD. The role of the 16p13.11 duplication in OCD, as well as multiple other psychiatric disorders, warrants further study. Although the mechanisms by which pdCNV contribute to OCD and/or CTD phenotype is not readily apparent at this point, multiple studies have demonstrated that pdCNV are a predictor of medical and neurodevelopmental disorders. CNV testing in those with OCD or CTD will help disentangle genotype-phenotype correlations and could lead to targeted therapeutics or treatment stratification. With further studies, the presence of pdCNV or other damaging genetic variation may provide clinicians with valuable information for predicting the trajectories of these neurodevelopmental disorders as well as the likelihood of co-occurring medical and psychiatric conditions. 

## Figures and Tables

**Table 1:** Characterization of the OCD and CTD probands.

	Total (*n*, %)	Average Diagnosis Age (SD)	Diagnosed < 18 (% of Total)	Diagnosed ≥ 18 (% of Total)
OCD Probands	993 ^1^	21.9 (7.0)	311 (31%)	681 (69%)
Female	623 (63%) ^2^	22.0 (6.8)	178 (29%)	444 (71%)
Male	370 (37%) ^2^	21.5 (7.4)	133 (36%)	237 (64%)
CTD Probands	217 ^3^	17.5 (6.9)	149 (69%)	67 (31%)
Female	77 (36%)	17.0 (6.7)	49 (64%)	27 (36%)
Male	140 (64%)	17.8 (7.1)	100 (71%)	40 (29%)

^1^ Seven individuals had missing values for diagnosis age. ^2^ Diagnosis age was missing for one individual. ^3^ Sex variable was missing for one individual SD: standard deviation.

**Table 2:** Characterization of the OCD and CTD probands with potentially damaging CNV.

	Probands with pdCNV (%)	Probands with pdCNV and Diagnosed < 18 (% Diagnosed < 18)	Probands with pdCNV and Diagnosed ≥ 18 (% Diagnosed ≥ 18)
OCD Probands	86 (9%)	27 (9%)	59 (9%)
Female	51 (8%)	15 (8%)	36 (8%)
Male	35 (9%)	12 (9%)	23 (10%)
CTD Probands	18 (8%)	13 (9%)	5 (7%)
Female	8 (10%)	6 (12%)	2 (7%)
Male	10 (7%)	7 (7%)	3 (8%)

The percentages for each category are the number of pdCNV carriers divided by the number of all individuals in that category. pdCNV: potentially damaging copy number variation.

**Table 3:** Known genomic disorders identified.

ID	Sex	Chromosomal Disorder	OCD, Diagnosis Age	CTD, Diagnosis Age	Co-Occurring Psychiatric Conditions
1	Female	16p13.3 deletion (includes CREBBP)	Yes, 44	No	Bipolar disorder
2	Female	22q11.2 deletion (Velo-cardio-facial syndrome/DiGeorge syndrome; proximal, A-D; includes TBX1)	Yes, 34	No	None
3	Female	16p13.11 deletion (BP2-BP3; includes MYH11)	No	Yes, 15	None
4	Female	16p11.2 deletion (proximal, BP4-BP5; includes TBX6)	Yes, 24	No	Major depression, bulimia nervosa, specific (isolated) phobias
5	Male	15q25.2 deletion (LCR B-C, proximal)	Yes, 14	Yes, 14	Asperger’s syndrome
6	Male	1q21.1 deletion (BP3-BP4, distal; includes GJA5)	Yes, 23	No	None
7	Female	17q12 duplication (RCAD syndrome; includes HNF1B)	No, 24	Yes	Agoraphobia with panic disorder
8	Male	22q11.2 duplication (DGS/VCFS; proximal, A-D; includes TBX1)	Yes, 18	No	None
9	Male	5q35 duplication (Sotos syndrome; includes NSD1)	Yes, 17	No	ADHD, Asperger’s syndrome
10	Female	Xq28 duplication (int22h1/int22h2-flanked; includes RAB39B)	Yes, 31	No	Specific (isolated) phobias
11	Female	Xq28 duplication (int22h1/int22h2-flanked; includes RAB39B)	Yes, 13	No	None
12 *	Female	16p13.11 recurrent microduplication (neurocognitive disorder susceptibility locus)	Yes, 23	No	Major depression, bulimia nervosa
13 *	Male	16p13.11 recurrent microduplication (neurocognitive disorder susceptibility locus)	Yes, 33	No	None

* Not included in the downstream analysis.

**Table 4:** Co-occurring psychiatric conditions in all probands and probands with potentially damaging CNV.

Co-Occurring Psychiatric Condition	OCD Probands ^1^		CTD Probands ^2^		OCD with CTD Probands	
	Total (%) ^3^	With pdCNV (%) ^4^	Total (%) ^3^	With pdCNV (%) ^4^	Total (%) ^3^	With pdCNV (%) ^4^
ADHD	69 (7%)	6 (9%)	58 (27%)	3 (5%)	32 (35%)	2 (6%)
Anxiety disorders ^5^	216 (22%)	15 (7%)	29 (13%)	3 (10%)	18 (20%)	1 (5%)
ASD	48 (5%)	7 (15%)	26 (12%)	5 (19%)	14 (15%)	3 (21%)
Bipolar disorder	32 (3%)	4 (13%)	3 (1%)	0 (0%)	2 (2%)	0 (0%)
Borderline personality disorder	19 (2%)	0 (0%)	1 (<1%)	-	1 (1%)	0 (0%)
Eating disorders	49 (5%)	3 (6%)	5 (2%)	0 (0%)	3 (3%)	0 (0%)
Major depression	199 (20%)	13 (7%)	36 (17%)	3 (8%)	22 (24%)	2 (9%)
Schizophrenia	8 (<1%)	0 (0%)	0 (0%)	-	0 (0%)	-

^1^ Six missing values. ^2^ One missing value. ^3^ Percentage of probands with the co-occurring psychiatric condition. ^4^ Percentage of probands with the co-occurring psychiatric condition with pdCNV. ^5^ Phobic anxiety disorders (F40) and other anxiety disorders (F41). ADHD: attention-deficit/hyperactivity disorder, ASD: autism spectrum disorder. OR: odds ratio, pdCNV: potentially damaging copy number variation.

**Table 5:** OCD and CTD probands with and without co-occurring psychiatric conditions.

	All Probands		Diagnosed < 18		Diagnosed ≥ 18	
	Total	Carriers of pdCNV (%)	Total	Carriers of pdCNV (%)	Total	Carriers of pdCNV (%)
OCD probands without CTD						
No co-occurring psychiatric condition	578	52 (9%)	174	17 (10%)	404	35 (9%)
At least one co-occurring psychiatric condition	324	26 (8%)	78	4 (5%)	246	22 (9%)
CTD probands without OCD						
No psychiatric co-occurring psychiatric condition	78	6 (8%)	50	3 (6%)	28	3 (11%)
At least one co-occurring psychiatric condition	48	4 (8%)	29	3 (10%)	19	1 (5%)

pdCNV: potentially damaging copy number variation.

**Table 6:** Odds ratios for co-occurring psychiatric conditions in carriers with potentially damaging CNV.

Phenotypes	OCD Probands			CTD Probands		
	OR	95% CI	*p*-Value	OR	95% CI	*p*-Value
ADHD	0.97	(0.36,2.16)	0.94	0.54	(0.12,1.71)	0.34
Anxiety disorders	0.75	(0.41,1.30)	0.33	1.26	(0.28,4.18)	0.74
ASD	1.80	(0.71,3.98)	0.17	3.46	(1.02,10.30)	0.03 *
Bipolar disorder	1.30	(0.31,3.82)	0.67	-	-	-
Eating disorders	0.71	(0.17,2.04)	0.58	-	-	-
Major depression	0.70	(0.36,1.24)	0.25	0.99	(0.22,3.22)	0.99

There were not enough CTD cases to estimate OR for bipolar disorder, borderline personality disorder, eating disorder, and schizophrenia. There were not enough OCD cases to estimate OR for borderline personality disorder and schizophrenia. ADHD: attention-deficit/hyperactivity disorder, ASD: autism spectrum disorder, OR: odds ratio. Significance level: * *p* < 0.05.

**Table 7:** Severity of OCD symptoms based on OCI-R score for probands with and without potentially damaging CNV.

	Intercept	pdCNV	TimeD	Sex	pdCNV × TimeD	Marginal Mean without pdCNV n = 502		Marginal Means with pdCNV n = 49	
						Mean	95% CI	Mean	95% CI
Total score	28.2 *	−4.6	−1.2 *	2.1	0.99	21.5	(20.3,22.7)	23.6	(19.8,27.4)
Washing	5.5 *	1.3	−0.3 *	0.8 *	−0.08	3.8	(3.5,4.1)	4.6	(3.6,5.6)
Obsessing	6.4 *	−1.2	−0.2 *	0.2	0.2	3.8	(3.5,4.1)	4.6	(3.6,5.6)
Hoarding	2.7 *	−0.2	−0.1 *	−0.2	0.1	2.0	(1.7,2.2)	2.2	(1.5,2.9)
Ordering	4.8 *	−3.2^*^	−0.2 *	0.6 *	0.6 *	3.7	(3.4,4.0)	4.2	(3.2,5.1)
Checking	5.2 *	−3.3^*^	−0.2 *	0.5	0.5 *	4.2	(3.8,4.4)	4.2	(3.3,5.2)
Neutralizing	3.7 *	2.0	−0.2 *	0.2	−0.2	2.7	(2.4,3.0)	3.1	(2.1,4.1)

pdCNV: potentially damaging copy number variation, TimeD: the difference between the older diagnosis date in the National Patient Register and the date the individual completed the OCI-R questionnaire, measured in years (we refer to it as time difference). Significance level: * *p* < 0.05.

## Data Availability

Study data are maintained at the Department of Medical Epidemiology and Biostatistics, Karolinska Institutet, Stockholm, Sweden. Extracted DNA samples are stored at Karolinska Biobank [https://ki.se/en/research/ki-biobank, accessed on 2 August 2022)] Biological samples can be made available to approved researchers.

## Supplementary Materials

The following supporting information can be downloaded at: https://www.mdpi.com/article/10.3390/genes13101796/s1, Table S1: List of genomic disorders derived from ClinGen and DECIPHER; Table S2: List of potentially damaging CNV.
